# Rational Improvement of Rice Yield and Cold Tolerance by Editing the Three Genes *OsPIN5b*, *GS3*, and *OsMYB30* With the CRISPR–Cas9 System

**DOI:** 10.3389/fpls.2019.01663

**Published:** 2020-01-09

**Authors:** Yafei Zeng, Jianyu Wen, Weibo Zhao, Qiong Wang, Wenchao Huang

**Affiliations:** State Key Laboratory of Hybrid Rice, Key Laboratory for Research and Utilization of Heterosis in Indica Rice, The Yangtze River Valley Hybrid Rice Collaboration & Innovation Center, College of Life Sciences, Wuhan University, Wuhan, China

**Keywords:** *OsPIN5b*, *GS3*, *OsMYB30*, cold tolerance, panicle length, grain length, CRISPR/Cas9

## Abstract

Significant increases in rice yield and stress resistance are constant demands for breeders. However, high yield and high stress resistance are often antagonistic to each other. Here, we report several new rice mutants with high yield and excellent cold tolerance that were generated by simultaneously editing three genes, *OsPIN5b* (a panicle length gene), *GS3* (a grain size gene) and *OsMYB30* (a cold tolerance gene) with the CRISPR–Cas9 (clustered regularly interspaced short palindromic repeats-associated protein 9) system. We edited two target sites of each gene with high efficiency: 53% for *OsPIN5b*-site1, 42% for *OsPIN5b*-site2, 66% for *GS3*-site1, 63% for *GS3*-site2, 63% for *OsMYB30*-site1, and 58% for *OsMYB30*-site2. Consequently, the *ospin5b* mutants, the *gs3* mutants, and the *osmyb30* mutants exhibited increased panicle length, enlarged grain size and increased cold tolerance, respectively. Then nine transgenic lines of the *ospin5b/gs3*, six lines of *ospin5b/osmyb30* and six lines of *gs3/osmyb30* were also acquired, and their yield related traits and cold tolerance corresponded to the genes being edited. Additionally, we obtained eight *ospin5b/gs3/osmyb30* triple mutants by editing all three genes simultaneously. Aside from the *ospin5b/gs3/osmyb30-4* and *ospin5b/gs3/osmyb30-25* mutants, the remaining six mutants had off-target events at the putative off-target site of *OsMYB30*-site1. The results also showed that the T_2_ generations of these two mutants exhibited higher yield and better cold tolerance compared with the wild type. Together, these results demonstrated that new and excellent rice varieties with improved yield and abiotic stress resistance can be generated through gene editing techniques and may be applied to rice breeding. Furthermore, our study proved that the comprehensive agronomic traits of rice can be improved with the CRISPR–Cas9 system.

## Introduction

Rice is one of the most important cereal crops in the world and feeds more than half of the world’s population (Khush, 1999). However, a shortage of rice has been an urgent threat for breeders as the human population has continued expanding for several decades (Wang and Li, 2008; Xing and Zhang, 2010). Rapid changes of climate have further restricted the rice yield. Therefore, it is an urgent matter for breeders to adopt advanced breeding methods that can not only improve the rice yield but also make rice more environmentally adaptable.

Precise gene engineering technologies, such as the transcription activator-like effector nucleases (TALENs) (Boch and Bonas, 2010), zinc finger nucleases (ZFNs) (Kim et al., 1996) and CRISPR–Cas9 (Jinek et al., 2012), have become fashionable strategies for developing new elite plant varieties to alleviate food insecurity owing to their high efficiency, speed and accuracy (Li et al., 2016; Li et al., 2017; Shen et al., 2018). Among those fashionable strategies, the CRISPR–Cas9 system is the most popular method to solve the outstanding issues currently because of its well-researched and constant improvement (Cong et al., 2013; Li et al., 2013; Mali et al., 2013).

In the past several decades, traditional hybrid breeders have been troubled by the cultivation of hybrid crops for the reason that their progeny did not have stable maintenance of their beneficial phenotypes owing to genetic segregation. Recently, researchers have made it possible to retain parental heterozygosity by the subsequent generations clonally through seeds using the CRISPR–Cas9 system. In their studies, clonal progeny was obtained through seeds when the edited *BBM1*, *BBM2* and *BBM3* genes substituting mitosis for meiosis (MiMe) and the expression of *BBM1* are combined in the egg cell (Khanday et al., 2019). Researchers also edited four genes, *REC8*, *PAIR1*, *OSD1* and *MTL,* in hybrid rice for clonal reproduction of F_1_ rice hybrids through seeds (Wang et al., 2019). Therefore, the extensive usage of this system provides new scientific guidance for adopting advanced techniques to develop elite rice varieties.

Rice yield is related to many agricultural traits: panicle number/plant, grain number/panicle, thousand grain weight and so on. Many important agricultural trait-related genes provide valuable resources for the application of bioengineering methods to improve the quality of crops. For example, OsPIN5b, an endoplasmic reticulum (ER) localized protein, has been inferred to be an auxin carrier and has important functions in auxin balance and transport. The expression of *OsYUC1*, an IAA related gene, is significantly increased in the *OsPIN5b* overexpression plants. Decreased expression of *OsPIN5b* leads to pleiotropic effects characterized as longer panicles, higher tiller number and higher rice yield compared with the wild type (Lu et al., 2015). GS3, which is widely used in the study of rice yield improvement, is comprised of four different functional domains that participate in the grain size regulatory network: an organ size regulation (OSR) domain, a transmembrane domain, a tumor necrosis factor receptor/nerve growth factor receptor (TNFR/NGFR) family cysteine-rich domain and a von Willebrand factor type C (VWFC) domain. Functional loss of the OSR domain leads to long grains, while changing the structure of the TNFR/NGFR and VWFC domains produces the opposite effect (Mao et al., 2010).

Furthermore, rice yield can be affected by many environmental factors. For instance, the rice growth and yield can be influenced by cold stress, which is one of the important environmental factors. To date, several cold stress-related genes have been cloned, including *COLD1*, *OsSRFP1*, and *SGD1* (Fang et al., 2015; Ma et al., 2015b; Wang et al., 2017). Another cold-responsive R2R3-type MYB gene *OsMYB30*, can bind to the promoters of β-amylase (*BMY*) genes as a transcription factor and has a negative influence in rice cold tolerance. Under cold stress, OsMYB30 and OsJAZ9 function as a complex to suppress the expression of *BMY* genes, thus affecting the starch degradation and maltose accumulation, which may contribute to an increase in cold sensitivity (Lv et al., 2017). Because of their effects on rice yield, cold tolerance related genes should be given more attention for their application in coping with cold disasters and in the expansion of rice cultivation areas into the subtropical zone.

Here, we report several novel rice mutants produced by editing *OsPIN5b*, *GS3* and *OsMYB30* with the CRISPR–Cas9 system in Nipponbare, which is a typical japonica rice with its whole genome sequence available and is widely used as a donor in rice breeding (International Rice Genome Sequencing, 2005). Our study revealed that the *ospin5b* mutants, the *gs3* mutants and the *osmyb30* mutants exhibited increased panicle length, enlarged grain size and enhanced cold tolerance, respectively. When two of the three genes were edited, the transgenic plants had the corresponding phenotypes, proving that we could edit three genes simultaneously to obtain the new elite varieties. The results then showed that the two triple mutants, *ospin5b/gs3/osmyb30-4* and *ospin5b/gs3/osmyb30-25*, in which all three genes were edited simultaneously without off-target events, exhibited higher yield and better cold tolerance compared to the wild type. In summary, we provided an example of how to improve the comprehensive agricultural traits of rice through use of the CRISPR–Cas9 system, which may offer new possibilities for rice breeding.

## Materials and Methods

### Plant Materials

Nipponbare was used for rice transformation in this research. These materials under natural field conditions were grown in the Wuhan experimental fields of Wuhan University to investigate the yield related traits. The seedlings used for cold stress treatments were grown in a greenhouse or a chamber at 4°C.

### Vector Construction

All of the vectors were constructed as previously described (Ma et al., 2015a; Ma and Liu, 2016). First, the target adaptors were cloned into the pYLgRNA-OsU6a vector by using T4 DNA ligase. Then, the ligated products as a template were amplified using U-F and gR-R primers (**Table S2**). Next, secondary PCR was employed to ligate the six cassettes. Finally, *BsaI* and T4 DNA ligase were used to clone the cassettes into the pYLCRISPR/Cas9P_ubi_-H vector.

### Off-Target Detection

The potential off-target sites were predicted on the website http://crispr.hzau.edu.cn/CRISPR/. The primers (**Table S2**), designed from Primer premier 5, were used for amplifying the off-target sequences. Then, the PCR products were sequenced for the detection of off-target events.

### Measurements of Rice Traits

Plant height, panicle number/plant, grain number/panicle, pollen fertility, seed-setting rate and heading date were measured with three random T_2_ generation plants. Ten randomly grains from each plant were selected to measure the grain length and three T_2_ generation plants repeats. Grain weight was calculated with 1,000 grains and three repeats.

### Cold Stress Treatments

The cold stress treatments were performed as previous studies (Lv et al., 2017). The wild type and transgenic lines were grown in a greenhouse with normal environment until three-leaf stage, and then the all seedlings were transferred into a chamber at 4°C. After 5 to 10 days, the seedlings were transferred back to the normal environment to record their survival rates.

### RNA Extraction and qRT-PCR

Total RNA of the wild type and transgenic lines were extracted with TRIzol reagent according to the product manual. Then, the extracted RNA was treated and purified with chloroform and DNase I, and the purified RNA was used to synthesize cDNA using M-MLV (Invitrogen). Quantitative reverse transcription–PCR (qRT-PCR) was performed with the primers (**Table S2**) using the Lightcycler 480 (Roche), taking the *actin* gene as the control.

### Selection of Target Sequences

Target sequences were predicted on the website http://crispr.hzau.edu.cn/CRISPR/ and the secondary structure between the target sequence and sgRNA were predicted on the website http://unafold.rna.albany.edu/?q=mfold/RNA-Folding-Form2.3. We designed two sgRNAs for *OsPIN5b* (*Os08g0529000*), two for *GS3* (*Os03g0407400*), and two for *OsMYB30* (*Os02g0624300*) with the primers (**Table S2**).

### Rice Transformation

The CRISPR/Cas9 vector was transformed into *A. tumefaciens* strain EHA105 by electroporation. Transformation of rice was performed using a method described previously (Hiei et al., 1994). The genomic DNA was extracted from the T_0_ generation and the target sites were amplified by PCR (**Figure S1**) with the primers (**Table S2**). The PCR products were sequenced for the detection of mutations.

### Homology Modeling

The relative protein structures were modeled by SWISS-MODEL. Amino acid sequences of the wild type and mutants were imported to the predict website for modeling the optimum protein structures (**Figure S2**). According to the GMQE (Global Model Quality Estimation) values and the QMEAN4 scores, we selected 5yzg.1.L and 5a1s.1.B as optimum templates for OsPIN5b and OsMYB30, respectively. The predicted protein structures were analyzed by Swiss-PdbViewer software.

## Results

### The CRISPR–Cas9 System Had High Efficiency in Editing Three Genes in Rice

**Figure 1:**
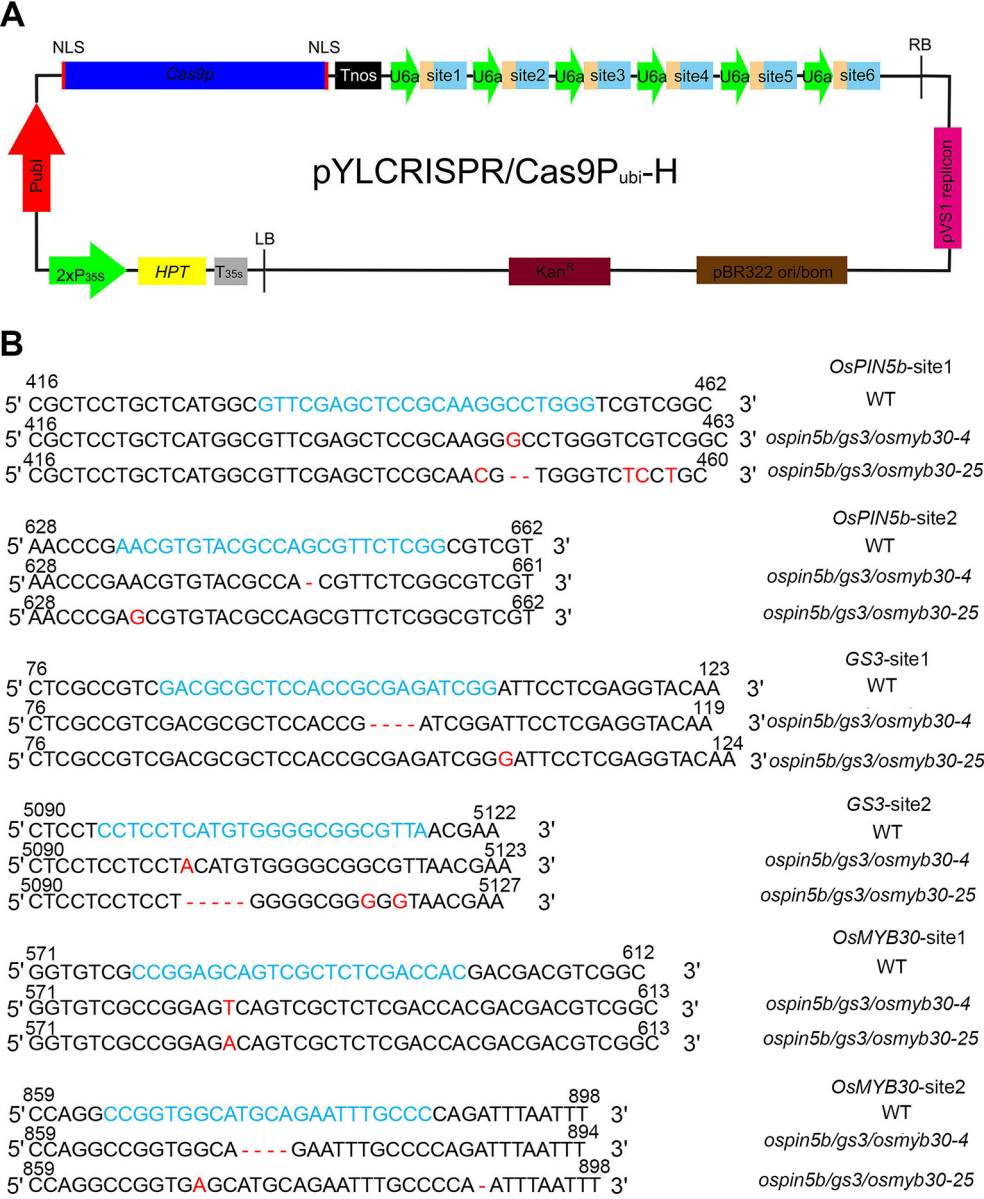
The *ospin5b/gs3/osmyb30-4* and *ospin5b/gs3/osmyb30-25* were precisely edited with CRISPR/Cas9 system. **(A)** Schematic diagram of the CRISPR/Cas9 vector. The pYLCRISPR/Cas9 binary vector was based on the pCAMBIA1300 backbone which contained the *Kanamycin* resistance gene. *HPT* encoded hygromycin B phosphotransferase, which could be driven by the cauliflower mosaic virus 35S promoter (P_35S_). The *Cas9* was driven by the maize ubiquitin promoter (P_ubi_) and used to edit target sites. Tnos was the terminator of nopaline synthase gene which was chose to terminate the expression of *Cas9* gene. U6a, small nuclear RNA promoters, was employed to facilitate the expression of multiple sgRNA cassettes. The six sgRNA cassettes also were inserted behind U6a. **(B)** Six target sequences alignment in *ospin5b/gs3/osmyb30-4* and *ospin5b/gs3/osmyb30-25*. Blue represents the target sequences. Red represents the changing base.

To obtain rice mutants with both high yield and good cold tolerance, we selected these three genes to edit: the panicle length gene *OsPIN5b*, the grain size gene *GS3* and the cold tolerance gene *OsMYB30*. Two target sites of each gene were selected: *OsPIN5b*-site1 and *OsPIN5b*-site2, *GS3*-site1 and *GS3*-site2, and *OsMYB30*-site1 and *OsMYB30*-site2 (**Figure S1**). We used Golden Gate assembly to clone six sgRNA cassettes into the CRISPR/Cas9 vector; the structure of the CRISPR/Cas9 vector was shown in **Figure 1A**. By examination of the PCR products of the target sites, we finally obtained a total of thirty-eight independent transgenic lines. The results showed that the CRISPR/Cas9 system had a high editing efficiency in the T_0_ generation: 53% for *OsPIN5b*-site1, 42% for *OsPIN5b*-site2, 66% for *GS3*-site1, 63% for *GS3*-site2, 63% for *OsMYB30*-site1, and 58% for *OsMYB30*-site2 (**Table 1**). A high frequency of homozygous or biallelic mutations were also found after analyzing the results with the Degenerate Sequence Decoding (DSD) website http://dsdecode.scgene.com/ (Liu et al., 2015). Finally, we identified eight triple mutants (*ospin5b/gs3/osmyb30*) in which all three genes were edited with high efficiency (21%) from these thirty-eight independent transgenic lines (**Table 1**). Thus, these studies demonstrated that the CRISPR/Cas9 system has high editing efficiency in manipulating multiple agronomic trait-related genes simultaneously.

**Table 1:** The editing efficiency of CRISPR/Cas9 system in rice.

**Target Site**	**No. of Independent Plants**	**No. of plants with mutation**			**Mutation rate (%)**
*OsPIN5b*-Site1	38		20	53	
*OsPIN5b* –Site2	38		16	42	
*GS3*-Site1	38		25	66	
*GS3*-Site2	38		24	63	
*OsMYB30*-Site 1	38		24	63	
*OsMYB30*-Site 2	38		22	58	
**Target gene**	**No. of independent plants**		**No. of plants with mutation**	**Mutation rate (%)**	
*OsPIN5b*	24		38	63	
*GS3*	25		38	66	
*OsMYB30*	26		38	68	
*OsPIN5b/GS3/OsMYB30*	8		38	21	

### *OsMYB30*-site1 Had Off-Target Events

Previous studies have demonstrated that off-target events are unavoidable when using the CRISPR/Cas9 system (Lawrenson et al., 2015). We wondered if there might have been off-target events that may disturb the comprehensive phenotypes in the eight *ospin5b/gs3/osmyb30* mutants. First, we selected two putative off-target sites for each target on the website http://crispr.hzau.edu.cn/CRISPR/. By amplifying the putative off-target sites of the eight triple-mutant lines, we found that only two of them, *ospin5b/gs3/osmyb30-4* and *ospin5b/gs3/osmyb30-25*, did not experience off-target events and that the remaining six had off-target events at the first putative off-target sites of *OsMYB30*-site1 (**Figure 1B**, **Table 2**). It was unexpected that the off-target rate of the first putative off-target site of *OsMYB30*-site1 was higher than that of the other putative off-target sites. Previous studies have put forward that mismatches between the seed sequence (12bp approach to the PAM) and the target sequence are important for decreasing the off-target frequency (Tsai et al., 2015). Sequence alignment of the twelve putative off-target sites showed that the most frequent off-target site had a higher similarity to its target site in both the seed sequence and target sequence compared with the other putative off-target sites. These results suggest that we should select target sites with low sequence similarity to other genes in the seed sequence and carefully target sequences to decrease the frequency of off-target events.

**Table 2:** The putative off-target event in the *ospin5b gs3 osmyb30* triple mutant lines.

Target Site	Putative off-target site	The sequence of putative off-target site	No. of plants	No. of plants with mutations	Mutation rate (%)
*OsPIN5b*-Site1	Chr3:9566074–9566052	CCTCGAGCTCTGCAAGGCTTTGG	8	0	0
	Chr1:19390027–19390049	TTTCGAGCTGCGCAAGGCGCGGG	8	0	0
*OsPIN5b*-Site2	Chr7: 8747238–8747260	CTCGTGTGCGCCAACGTTCTCGG	8	0	0
	Chr3:24251589–24251567	AACGTGCTCGTCAGCGTCCTCGG	8	0	0
*GS3*-Site 1	Chr8: 2495427–2495449	GACGCGCTCCACCGCGCGCTCGG	8	0	0
	Chr7:14816697–14816719	AACTCGCTCAACCGCGAGAGGGG	8	0	0
*GS3*-Site 2	Chr9: 1832101–1832123	TCGCGCCACCCCACACGAGGTGG	8	0	0
	Chr7: 27472889–27472911	TAACCCCGCACCGCATGAGGCGG	8	0	0
*OsMYB30*-Site 1	Chr3:13108929–13108951	GTGGCGGAGAGCGACTGCACGGG	8	6	75
	Chr5: 1475321–1475343	CTGCGCGAGAGCGACTGCGCCGG	8	0	0
*OsMYB30*-Site 2	Chr4: 24538627–24538649	TGACAAATGCTGCATGCCATGGG	8	0	0
	Chr2:28909447–28909469	CGTCAAATTCTGCATGACAGTGG	8	0	0

The green font represented the PAM motif (NGG); the red font represented the mismatch bases.

### The Panicle Length Was Obviously Increased in the *ospin5b* Mutants

**Figure 2:**
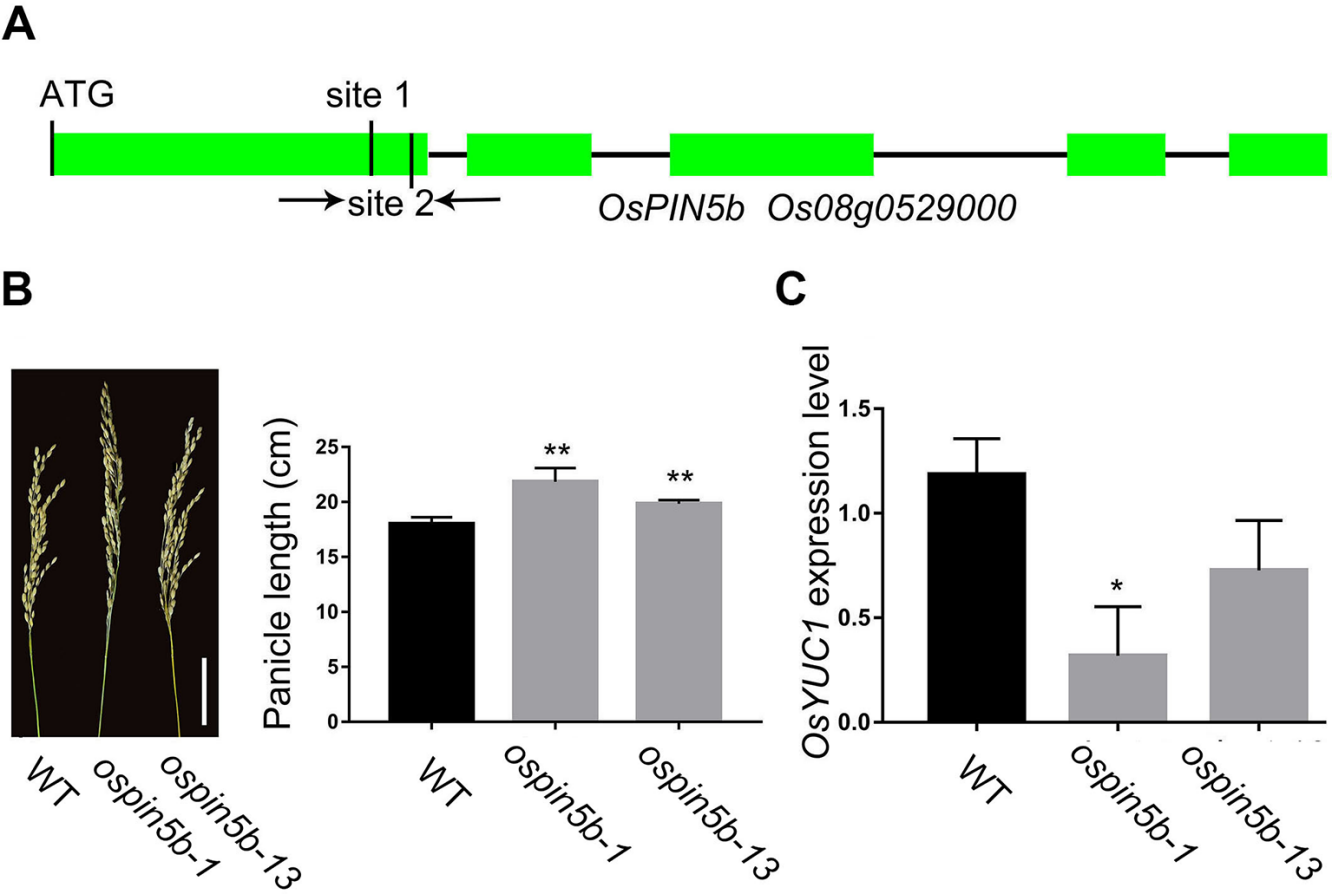
CRISPR/Cas9-induced *ospin5b* mutants and the expression level of cell cycle-related genes. **(A)** Schematic representation of the *OsPIN5b* genomic region. The two target sites of *OsPIN5b* and PCR primers were marked in first exon. **(B)** The phenotype of panicles and data statistics of *ospin5b-1*, *ospin5b-13* and WT, Bar = 5 cm. **(C)** The expression level of marker gene *OsYUC1* in WT, *ospin5b-1* and *ospin5b-13*. Data are means ± SD from three biological replicates. Student’s t test, **P < 0.001, *0.001 < P < 0.05.

OsPIN5b, located in the endoplasmic reticulum (ER), is involved in the balance and transport of auxin in rice (Lu et al., 2015). The *ospin5b* knockdown mutant showed more tiller numbers and longer panicles compared with the wild type. In this study, we selected two sites located in the first exon to change the coding region of *OsPIN5b* with the CRISPR–Cas9 system (**Figure 2A**). Finally, we identified 24 mutant lines of *OsPIN5b* from 38 independent lines (63%) (**Table 1**). T_2_ generation of the *ospin5b* mutants, *ospin5b-1* and *ospin5b-13*, in which only *OsPIN5b* had been edited, were planted in the field for further phenotype analysis. The average panicle length of the wild type is 18.3 cm, while the *ospin5b-1* and *ospin5b-13* mutants achieved 22.6 cm and 19.8 cm, respectively (**Figure 2B**).

Previous studies have proven that the expression level of many auxin biosynthesis and transport related genes in the ectopic expression *OsPIN5b* lines are different from that in the wild type. It is worth noting that the expression level of *YUCCA* genes and the content of IAA are affected in the ectopic expression lines. Therefore, we took *OsYUCCA1* as a marker gene to measure the effect of the CRISPR–Cas9 system *in vivo*. As expected, the expression level of the marker gene was significantly decreased in the *ospin5b-1* and *ospin5b-13* transgenic lines compared with the wild type (**Figure 2C**). Together, these results revealed that the editing efficiency of the CRISPR–Cas9 system was high in the *OsPIN5b* target sites, and the panicle length of the *ospin5b* mutants was obviously increased compared with the wild type.

### The Grain Size Was Strikingly Enlarged in the *GS3* Edited Lines

**Figure 3:**
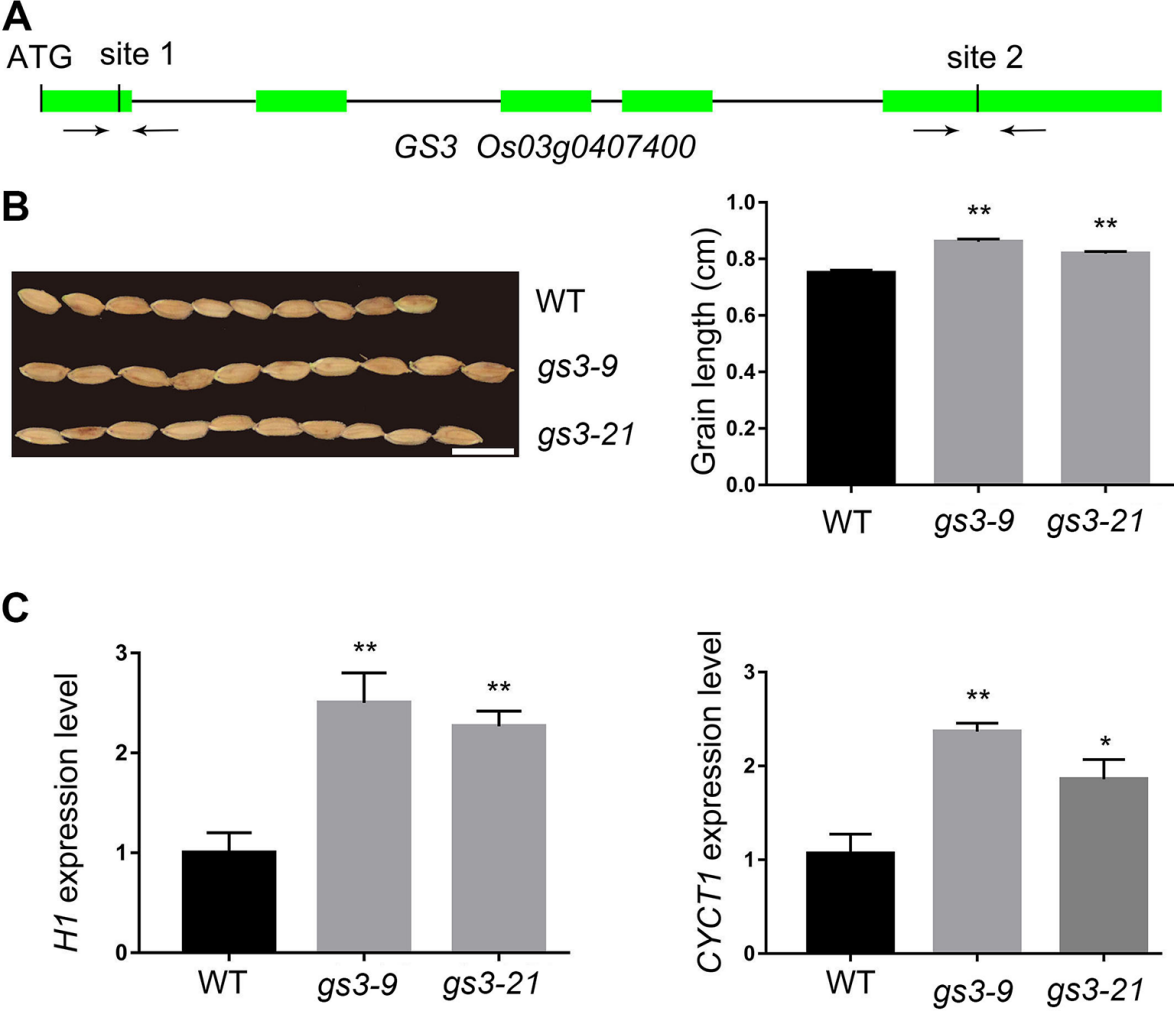
CRISPR/Cas9-induced *gs3* mutants and the expression level of cell cycle-related genes. **(A)** Schematic representation of the *GS3* genomic region. The two target sites of *GS3* and PCR primers were marked in first and fifth exons. **(B)** The grain phenotype of *gs3–9*, *gs3–21* and WT, and statistics for the average grain length. Bar = 1 cm. **(C)** The expression level of marker gene *OsH1* and *OsCYCT1* in WT, *gs3–9* and *gs3–21*. Data are means ± SD from three biological replicates. Student’s t test, **P < 0.001, *0.001 < P < 0.05.

The *GS3* gene has been cloned in the Minghui 63 background. A substitution of C to A in the allele of *GS3* resulted in a 178-amino-acid truncation and, thus, an altered grain size (Fan et al., 2006). In this study, aiming to interrupt the function of GS3, we chose two target sites from the website http://crispr.hzau.edu.cn/CRISPR/ to edit with the CRISPR–Cas9 system. The two sites are located in the first and fifth exons of *GS3* (**Figure 3A**). Finally, we acquired 25 *GS3* edited lines among the 38 independent transgenic lines (66%) (**Table 1**). In the 25 edited lines, some of the mutations were homozygous, which might happen before the embryogenic cell division (**Table S1**) (Zhang et al., 2014). T_2_ generation of the *gs3* edited lines, *gs3–9* and *gs3–21*, in which only *GS3* had been edited, were planted for grain length characterization. The average grain length of the wild type was 7.5 mm, while it was 8.6 mm of *gs3–9* and 8.2 mm of *gs3–21* (**Figure 3B**). In recent years, increasing numbers of genes have been cloned and studied, most of which regulate the grain size by controlling cell proliferation and expansion in spikelet hulls, such as *FZP*, *GS5*, and *GS2* (Li et al., 2011; Hu et al., 2015; Ren et al., 2018). Two genes, *CYCT1* and *H1*, involved in the cell cycle, were selected to measure the effects of the CRISPR/Cas9 system on the *GS3* target sites according to previous studies. The qRT-PCR results showed that the expression levels of the marker genes in *gs3–9* and *gs3–21* were significantly increased compared with the wild-type, which was consistent with expectations (**Figure 3C**). These results demonstrated that *GS3* was successfully edited with the CRISPR/Cas9 system, and the *gs3* mutants exhibited larger grain sizes in Nipponbare, which is consistent with previous studies (Fan et al., 2006).

### The *osmyb30* Mutant Lines Displayed Enhanced Cold Tolerance

**Figure 4:**
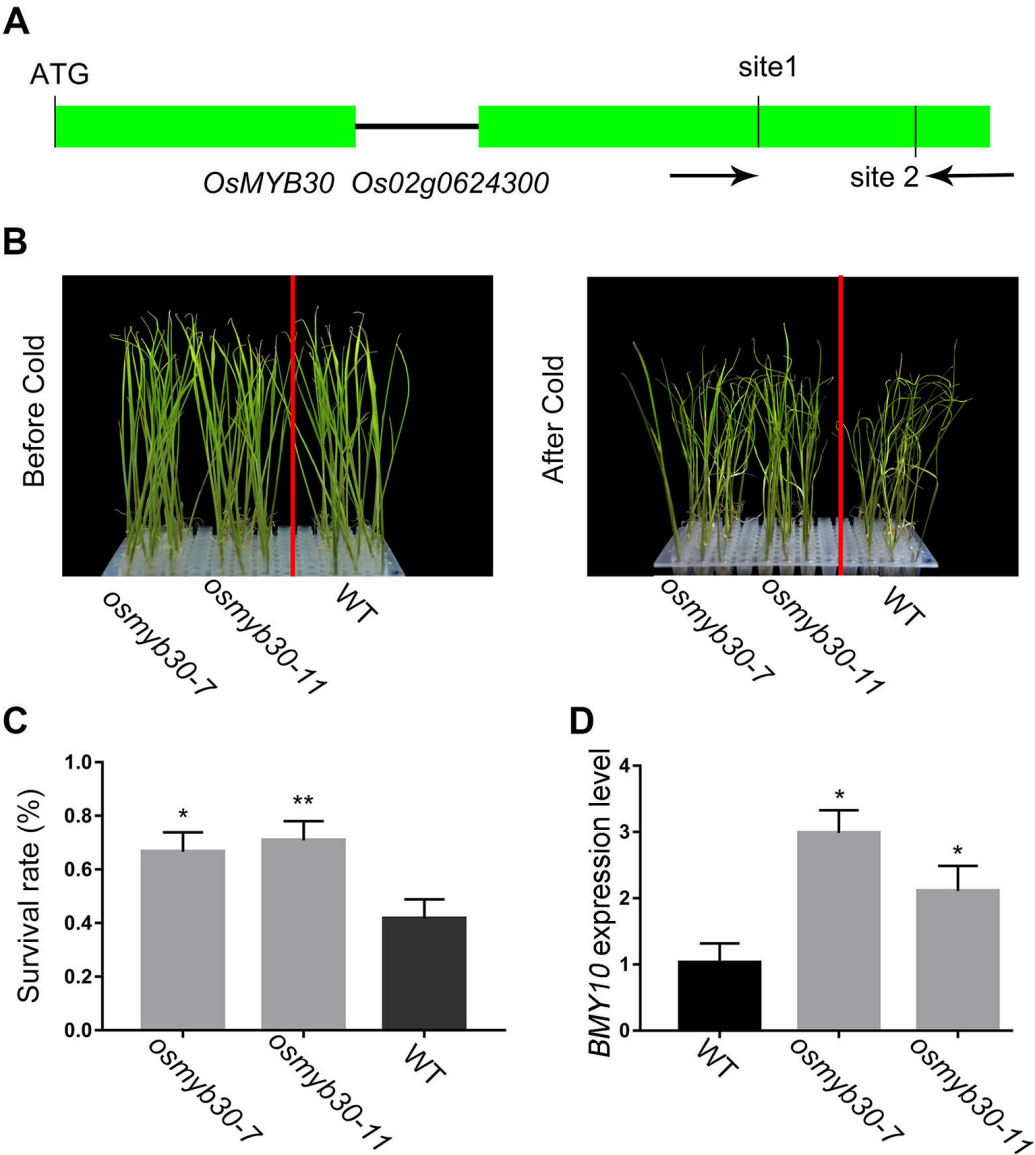
The phenotype analysis of *osmyb30* mutants and the expression level of downstream *BMY10* gene. **(A)** Schematic representation of the *OsMYB30* genomic region. The two target sites of *OsMYB30* and PCR primers were marked in second exon. **(B)** The seedlings of *osmyb30*-*7*, *osmyb30-11* and WT after being treated in 4°C chamber. **(C)** The survival rate of *osmyb30* mutants after 4°C treatment. **(D)** The expression level of marker gene *OsBMY10* in WT, *osmyb30-7* and *osmyb30-11*. Data are means ± SD from three biological replicates. Student’s t test, **P < 0.001, *0.001 < P < 0.05.

*OsMYB30* was characterized as a cold-responsive R2R3-type MYB gene. Previous studies showed that the *osmyb30* mutant, which was generated by inserting T-DNA in the second exon, exhibited weak cold sensitivity compared with the wild type and the overexpression lines. Therefore, we chose two target sites from the second exon of *OsMYB30* for editing with the CRISPR–Cas9 system (**Figure 4A**). Finally, 26 mutant lines were identified among the 38 independent lines, and the editing efficiency reached up to 68% (**Table 1**). Among the 26 mutant lines, most of them were single-base mutations, which belong to biallelic mutations (**Table S1**). T_2_ generation of the *osmyb30* mutants, *osmyb30-7* and *osmyb30-11*, in which only *OsMYB30* had been edited, were used to do the following experiments. Under the condition of 4°C, the wild type exhibited a wilting phenotype earlier than the two mutants *osmyb30-7* and *osmyb30-11* (**Figure 4B**), and the plant survival rates of *osmyb30-7* and *osmyb30-11* (66.7% and 70.8%, respectively) were higher than the wild type (41.7%; **Figure 4C**), indicating that the two mutant lines had better cold tolerance compared with the wild type. Previous study has proven that OsMYB30 is a transcription factor that interacts with OsJAZ9 to regulate the downstream *BMY* genes expression under a cold environment (Lv et al., 2017). Hence, we detected the expression level of *BMY10* in leaves grown at 4°C and found it was increased in the two mutant lines compared with the wild type (**Figure 4D**). Thus, the phenotypes of the *osmyb30* mutants indicated that the *OsMYB30* target sites were efficiently edited in rice, and the *osmyb30* mutant lines have similar effects in Nipponbare, which is consistent with the previous studies (Lv et al., 2017).

### The Phenotype Analysis of Double Mutants

**Figure 5:**
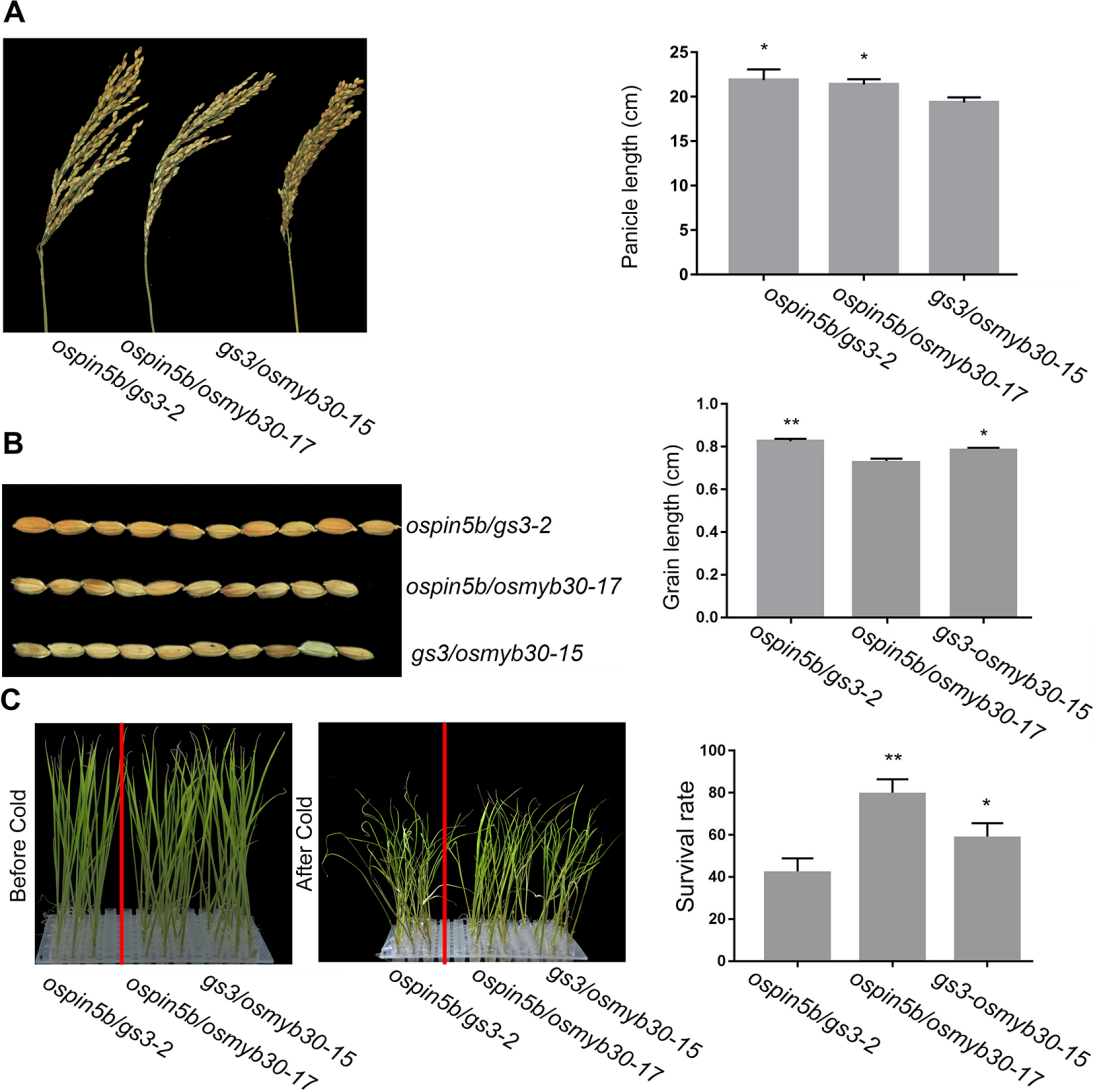
The phenotypes of *ospin5b/gs3-2*, *ospin5b/osmyb30-17* and *gs3/osmyb30-15* mutants. **(A)** The panicles and data statistics of *ospin5b/gs3-2*, *ospin5b/osmyb30-17* and *gs3/osmyb30-15* mutants, Bar = 5 cm. **(B)** The grain phenotype of *ospin5b/gs3-2*, *ospin5b/osmyb30-17* and *gs3/osmyb30-15*, and statistics for the average grain length. Bar = 1 cm. **(C)** The seedlings and survival rate of *ospin5b/gs3-2*, *ospin5b/osmyb30-17* and *gs3/osmyb30-15* after being treated in 4°C chamber. Data are means ± SD from three biological replicates. Student’s t test, **P < 0.001, *0.001 < P < 0.05.

When we detected the efficiency of the system, several double mutants of the three genes were identified: nine transgenic lines of the *ospin5b/gs3*, six lines of *ospin5b/osmyb30* and six lines of *gs3/osmyb30* (**Table 3**). To figure out whether the double mutants will show the corresponding phenotypes, *ospin5b/gs3*-2, *ospin5b/osmyb30*-17 and *gs3/osmyb30*-15 were chosen to measure their yield related traits and cold tolerance. As shown in **Table 4** and **Figure S3**, the panicle number/plant and grain number/panicle of the *ospin5b/gs3*-2 and *ospin5b/osmyb30*-17 double mutants were improved compared with the wild type and the *gs3/osmyb30*-15 mutants while the thousand grain weight of the *ospin5b/gs3*-2 and *gs3/osmyb30*-15 double mutants were increased compared with the wild type and the *ospin5b/osmyb30*-17 mutants. The three double mutants were transferred into a chamber at 4°C at the three-leaf stage to test the cold tolerance and the *ospin5b/osmyb30*-17 *and gs3/osmyb30*-15 showed a wilting phenotype later than *ospin5b/gs3*-2 (**Figure 5**). The plant survival rates of *ospin5b/osmyb30*-17 *and gs3/osmyb30*-15 (79.1% and 58.3%) were higher than *ospin5b/gs3*-2 (47.6%) as showed in **Figure 5**. The data of the three double mutants indicated that the traits could be added together in the same mutants and we could study the new and elite varieties with multiple genes were edited simultaneously.

**Table 3:** The number of single mutants, double mutants and triple mutants.

	*OsPIN5b*	*ospin5b*	*ospin5b/gs3*	*ospin5b/osmyb30*	*ospin5b/gs3/osmyb30*
No.	24	1	9	6	8
	*GS3*	*gs3*	*ospin5b/gs3*	gs3/osmyb30	*ospin5b/gs3/osmyb30*
No.	25	2	9	6	8
	*OsMYB30*	osmyb30	*ospin5b/osmyb30*	gs3/osmyb30	*ospin5b/gs3/osmyb30*
No.	26	6	6	6	8

**Table 4:** The phenotype of wild type and edited lines.

Characters	Plant Height (cm)	Panicle number/plant	Grain number/panicle	Thousand grain weight (g)	Yield/plant (g)	Pollen fertility (%)	Seed-setting rate (%)	Heading date (d)
Wild type	97.8 ± 1.0	9.7 ± 1.5	92.6 ± 6.0	23.8 ± 0.4	19.3 ± 2.0	100 ± 0	89.1 ± 1.7	77 ± 2.6
*ospin5b-1*	98.1 ± 1.3	13.6 ± 0.5*	115.0 ± 7.5*	23.5 ± 0.7	27.0 ± 1.7**	100 ± 0	89.6 ± 2.8	77 ± 0.5
*gs3*-9	97.7 ± 1.4	9.3 ± 1.1	90.6 ± 3.0	26.8 ± 0.3**	22.8 ± 1.3*	100± 0	87.0 ± 3.3	76 ± 1.5
*ospin5b/gs3-2*	97.6 ± 0.6	13.6 ± 0.5*	117.0 ± 7*	26.4 ± 0.2*	28.5 ± 0.7**	100 ± 0	89.4 ± 1.8	77 ± 0.3
*ospin5b/osmyb30-17*	98.3 ± 1.1	13.3 ± 1.1*	115.0 ± 6.9*	23.4 ± 1.3	27.0 ± 0.6**	100 ± 0	87.5 ± 0.5	77 ± 1.1
*gs3/osmyb30-15*	96.7 ± 0.4	10.3 ± 0.5	93.6 ± 5.5	25.2 ± 0.7*	22.8 ± 0.7	100 ± 0	89.6 ± 2.6	78 ± 1.5
*ospin5b/gs3/osmyb30-4*	97.3 ± 0.9	12.3 ± 0.5*	116 ± 4.5**	25.3 ± 0.4*	30.4 ± 0.9**	100 ± 0	90.4 ± 2.6	78 ± 0.5
*ospin5b/gs3/osmyb30-25*	99.1 ± 0.5	14.3 ± 1.1*	113.6 ± 7.2*	24.9 ± 0.3*	28.7 ± 2.3**	100 ± 0	89.7 ± 1.3	80 ± 1.1

Data are means ± SD from three biological replicates. Student’s t test, **P < 0.001, *0.001 < P < 0.05.

### The *ospin5b/gs3/osmyb30* Triple Mutants Exhibited High Yield and Good Cold Tolerance

**Figure 6:**
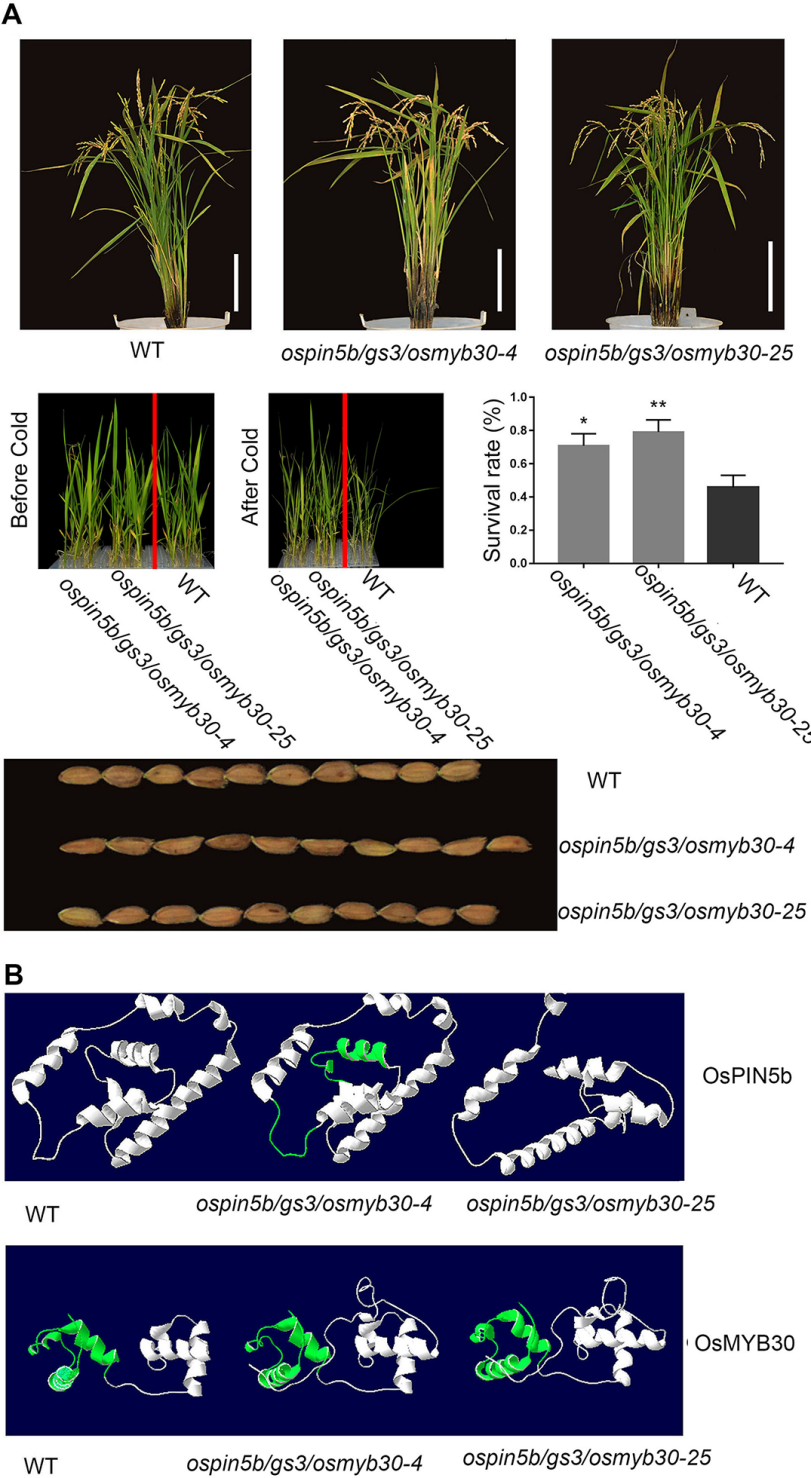
The phenotypes of *ospin5b/gs3/osmyb30*-*4*, *ospin5b/gs3/osmyb30-25* and the predicted protein structure of OsPIN5b and OsMYB30 in *ospin5b/gs3/osmyb30-4*, *ospin5b/gs3/osmyb30-25*. **(A)** Whole plant morphology, survival rate after cold treatment and grain size in WT, *ospin5b/gs3/osmyb30-4*, *ospin5b/gs3/osmyb30-25*. Bar = 20 cm. **(B)** The OsPIN5b and OsMYB30 protein structures of WT, *ospin5b/gs3/osmyb30-4* and *ospin5b/gs3/osmyb30-25* were predicted by SWISS-MODEL. The observed differential regions were highlighted in green.

We have generated the *ospin5b* mutant*, the gs3* mutant, and the *osmyb30* mutant with the CRISPR–Cas9 system, and their phenotypes were consistent with the previous studies. To investigate the phenotypes of plants with all three genes edited simultaneously, we obtained two triple mutants, named *ospin5b/gs3/osmyb30-4* and *ospin5b/gs3/osmyb30-25*, which did not experience off-target events (**Figure S2**). T_2_ generations of *ospin5b/gs3/osmyb30-4* and *ospin5b/gs3/osmyb30-25* had normal characteristics, including plant height, pollen fertility, and seed-setting rate, compared with the wild type (**Table 4**). However, they exhibited increased panicle length, less greenness, more tillers, larger grain size, and decreased cold sensitivity in the experimental field (**Figure 6A**). The yield-related traits are presented in **Table 4**, which showed the panicle number/plant, grain number/panicle and thousand grain weight. Under the condition of 4 °C, the plant survival rates of *ospin5b/gs3/osmyb30-4* and *ospin5b/gs3/osmyb30-25* were 70.8% and 79.1%, respectively, while that of the wild type was only 45.8% (**Figure 6A**).

The function of proteins is known to be based on their three-dimensional structures, so it was necessary for us to determine the structures of the mutants, which can be achieved by SWISS-MODEL. Therefore, we modeled the structures of OsPIN5b and OsMYB30 both in mutated form and original form, respectively. From **Figure 6B** we could clearly observe that there was a slight change in the distortion angle of the OsPIN5b protein in *ospin5b/gs3/osmyb30-4* and a completely different form in *ospin5b/gs3/osmyb30-25* compared with the wild type. For OsMYB30, the conserved Myb-like DNA-binding domain in the two mutants had a looser structure compared with that in the wild type (**Figure 6B**). Furthermore, the two mutated forms of OsMYB30 had other obvious changes compared with wild type OsMYB30 (**Figure 6B**). The mutations of *ospin5b/gs3/osmyb30-4* and *ospin5b/gs3/osmyb30-25* caused a truncation in the OSR domain of GS3, leading to the functional loss of this protein. Therefore, there was no need to predict its structures.

Taken together, we can conclude that the CRISPR–Cas9 system can efficiently edit the three genes alone or simultaneously. Breeders may be able to simultaneously manipulate multiple agriculture traits with this system to cultivate new varieties.

## Discussion

To date, the CRISPR/Cas9 system has been used to solve challenges that have not been overcome with traditional approaches in rice, such as the coordination between disease resistance and high yield. With the development of this technology, many genes in a good number of crops have been edited with this system, such as sorghum, maize, rice, wheat, soybean, potato, cotton, grapefruit, and watermelon (Shan et al., 2013; Zhang et al., 2016). In this study, we gained new rice mutants with both high yield and good cold tolerance by simultaneously editing the yield-related genes (*OsPIN5b* and *GS3*) and the cold stress related gene (*OsMYB30*) with the CRISPR–Cas9 system, which decreased many manpower and material resources compared with the traditional breeding method. We obtained single mutants, double mutants and triple mutants together to study the relationship of high yield and abiotic resistance, which has been an obstacle to phenotype improvement.

However, the CRISPR–Cas9 system has the weakness of unavoidable off-target events, and improving the accuracy of this system is a challenge that scientists have been working to solve. Here, we obtained eight transgenic plants where all three genes were edited simultaneously with the CRISPR–Cas9 system. However, six of them had off-target events at the first off-target site of *OsMYB30*-site1. By analyzing the sequence of these off-target sites, we suggest that the selected target sites should have a GC content of 50–70% with fewer than four consecutive Ts. Furthermore, those sites should avoid forming hairpin or stem structures with the sg-RNA sequences. In addition, they should contain more mismatches in the seed sequence and target sequence (Ma and Liu, 2016).

Nipponbare is a model plant that has been widely used and studied in rice breeding. Therefore, we adopted it as the material to study how to improve its shorter panicles and cold tolerance compared with other rice cultivars. Since yield and resistance related genes have pleiotropic effects on the phenotypes of rice in different rice varieties backgrounds (Fan et al., 2006), it was necessary to determine the effects of each single gene in Nipponbare before studying the comprehensive phenotypes of these three genes together.

First, the PIN protein family has 12 members in rice and most of them participate in the regulatory network of plant hormones, exhibiting functional redundancy (Xu et al., 2005; Wang et al., 2009; Zhang et al., 2012). In previous studies, the panicle length was significantly increased in *ospin5b* knockdown lines. In this study, the *ospin5b* mutants showed less greenness, which may affect the absorption of light energy and thus the yield, but the panicle number/plant and grain number/panicle in the mutants still increased (**Table 4**), indicating that the defect of less greenness was not enough to affect the yield. In addition, the PIN protein family has 12 members in rice and most of them participate in the regulatory network of plant hormone with exhibiting functional redundancy, so there were no significant changes of other agricultural traits in the knock out mutants.

Second, it was reported that GS3 negatively regulates grain length, and its nonsense mutant allele exists in many large grain rice varieties. In Nipponbare, the thousand grain weight and grain size were significantly increased in the *gs3* edited lines, while no other phenotypes changed significantly. This result indicated that the *GS3* gene has a positive impact on yield in the Nipponbare background.

Third, OsMYB30 is a nuclear protein and acts as a negative regulator of rice cold tolerance. In the previous study, survival rate of the mutant was significantly higher (80%) than the wild type of ZH11 (less than 20%) (Lv et al., 2017). In this study, the plant survival rates of *osmyb30-7* and *osmyb30-11* (66.7% and 70.8%, respectively) were higher than the wild type (41.7%) under the condition of 4 ℃. Compared with previous study, survival rates of the mutants in this study were not as high as previous study, possibly because the smaller seedlings (three-leaf stage seedlings) in this study was more cold sensitive than the seedlings (four-leaf stage seedlings) used in the previous study. Because the cold treatment was performed at the three-leaf stage, the surviving plants were not influenced in the following growth periods. Together, these results indicated that the CRISPR–Cas9 system can be used to improve rice yield by editing *OsPIN5b* and *GS3* and enhance cold tolerance by editing *OsMYB30* in Nipponbare.

Since the single mutants of the three genes had phenotypes consistent with those reported in previous studies and the other phenotypes were not influenced, we then identified the comprehensive phenotypes of the double mutants and triple mutants. After analyzing the phenotypes of double mutants, we concluded that these mutants showed the desired phenotypes which corresponded to the edited genes. The success of editing two genes together made us sure that we could edit three genes simultaneously to obtain the elite varieties. As shown in **Figure 6A**, **Table 4** and **Table S3**, several agricultural traits of the *ospin5b/gs3/osmyb30* triple mutants were improved, while the length of the grain did not achieve the desired effect like the single *gs3* mutants. We can attribute this phenomenon to the overlapping or influence of the three regulation networks, the incompatibility between high yield and high resistance, the limitations of production growth and so on. Nevertheless, we confirmed that the *ospin5b/gs3/osmyb30* triple mutants exhibited both higher yield and better cold tolerance compared with the wild type.

Together, these results indicated that the CRISPR–Cas9 system had high efficiency in editing these three genes both individually and simultaneously. At the same time, the successful transformation of phenotypes in *ospin5b/gs3/osmyb30-4* and *ospin5b/gs3/osmyb30*-25 has provided a new method to solve problems encountered in traditional rice breeding. According to our findings, we highly recommend that preferable rice materials should be obtained by rationally manipulating those genes which control different agricultural traits in rice.

## Conclusion

In this study, we successfully edited *OsPIN5b*, *GS3* and *OsMYB30* individually and simultaneously with high efficiency in rice through use of the CRISPR–Cas9 system. We obtained several novel rice mutants with high yield, good cold tolerance or both that may be applied to rice breeding. Additionally, our studies demonstrated that the simultaneous improvement of multiple agronomic traits can be achieved by the CRISPR–Cas9 system.

## Data Availability Statement

All datasets generated for this study are included in the article/**Supplementary Material**.

## Author Contributions

WH designed the study. YZ performed the entire experiment. JW performed vector construction and rice transformation. WZ and QW performed the RNA extraction.

## Funding

This research was supported by the National Key R&D Program of China (2017YFD0100400), National Natural Science Foundation of China (31771746) and the National Rice Industry Technology System (CARS-01-07).

## Conflict of Interest

The authors declare that the research was conducted in the absence of any commercial or financial relationships that could be construed as a potential conflict of interest.
