# Supplementary material for: Rational Improvement of Rice Yield and Cold Tolerance by Editing the Three Genes OsPIN5b, GS3, and OsMYB30 With the CRISPR–Cas9 System
Source: Front Plant Sci. 2020 Jan 9;10:1663. doi: 10.3389/fpls.2019.01663 (PMC6964726; doi:10.3389/fpls.2019.01663)
Supplement: Supplementary file 1 [file DataSheet_1.docx]

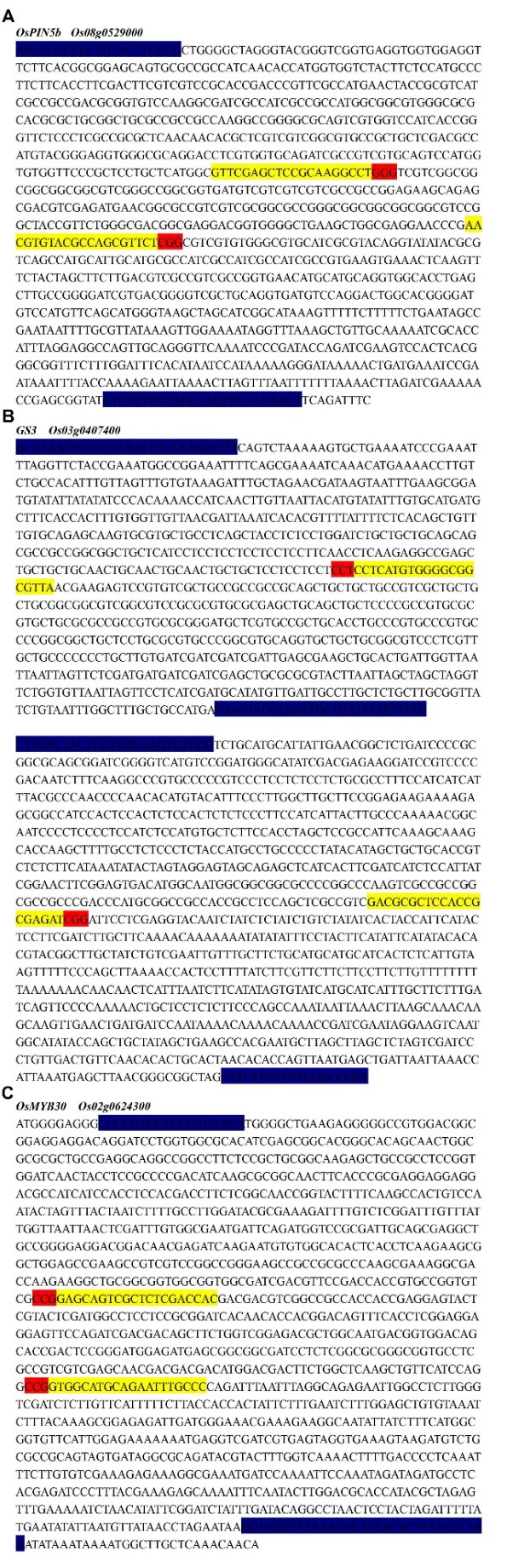


**Figure S1.** Target sites and adjacent genomic sequences of *OsPIN5b* (A), *GS3* (B) and *OsMYB30* (C). The red region, the PAM (NGG) sequences. The yellow region, the target sites. The blue regions, the relative primers used for identifying edit conditions.


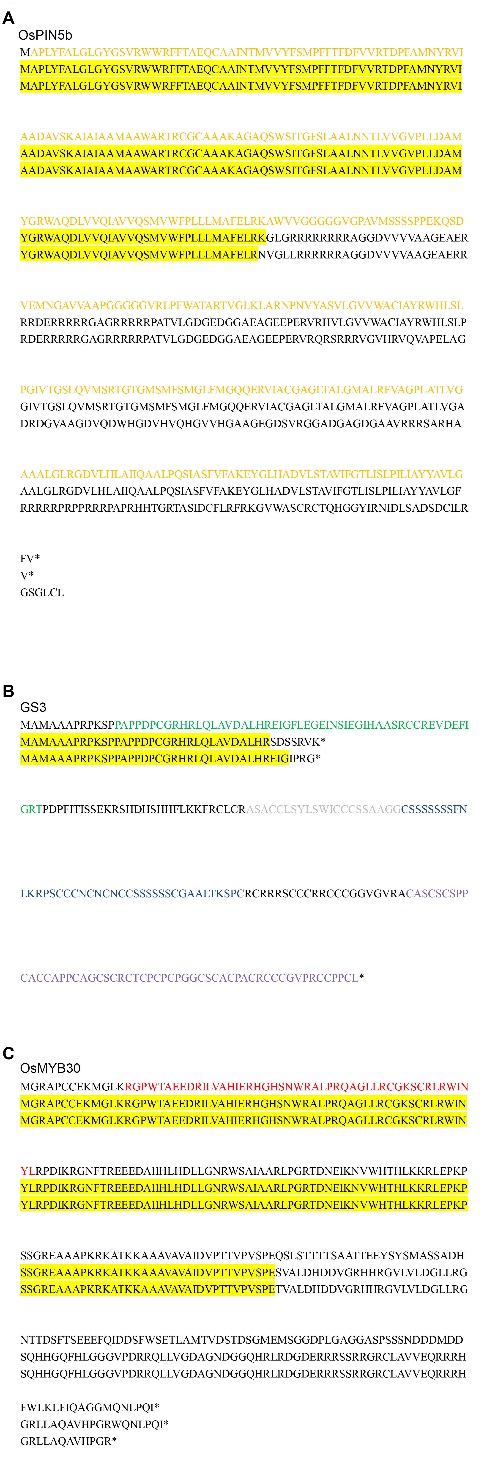


**Figure S2.** Amino acid sequences alignment of OsPIN5b (A), GS3 (B) and OsMYB30 (C). The same sequences of WT, plant-4, plant-25 were highlighted in yellow. Auxin Efflux Carrier (AEC) Family was highlighted in orange (A). The PEBP-like domain was highlighted in green. The transmembrane region was highlighted in gray. The TNFR/NGFR family cysteine-rich domain was highlighted in blue. The VWFC module was highlighted in purple (B) Myb-like DNA-binding domain was highlighted in red (C).


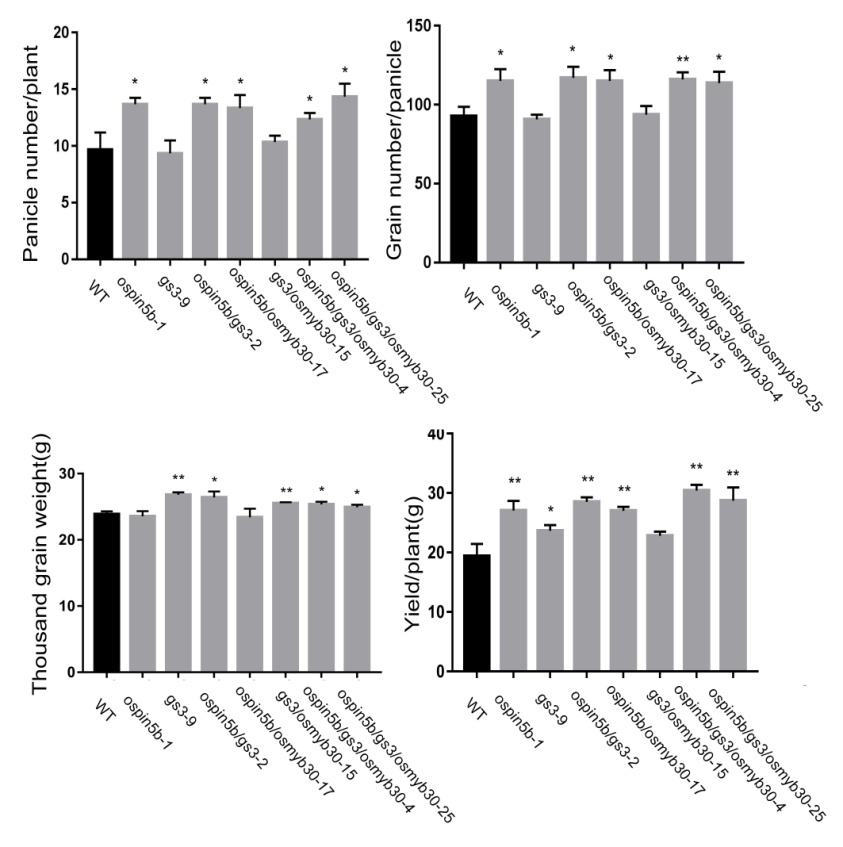


**Figure S3.** Statistical analysis of wild type and edited lines for the four yield-related traits. Data are means ±SD from three biological replicates. Student’s t test, **P < 0.001, *0.001 < P < 0.05.

**Table S1 Part of the sequence alignment of the target region in different mutant lines**

| *OsPIN5b*-site1 | 5’ GTTCGAGCTCCGCAAGGCCTGGG 3’ |  |
| --- | --- | --- |
| Plant 1 | 5’ GTTCGAGCTCCGCAAGGGCCTGGG 3’ | biallelic |
|  | 5’ GTTCGAGCTCCGCAAGGACCTGGG 3’ |  |
| Plant 13 | 5’ - - - - - - - - - - - - - - - - - AGTCCATGGTGTGGT 3’ | biallelic |
|  | 5’ - - - - - TC - - - - - - - - - - - -TCCATGGTGTGGT 3’ |  |
|  | 5’ GCAGATCGCCGTCGTGCAGTCCATGGTGTGGTTCCCGCTCCTGCTCATGG 3’ | Ref |
| Plant 5 | 5’ GTTCGAGCTCCGCAA - GCCTGGG 3’ | biallelic |
|  | 5’ GTTCGAGCTCCGCAA - - CCTGGG 3’ |  |
| Plant 32 | 5’ GTTCGAGCTCCGCAA - - CCTGGG 3’ | heterozygous |
|  | 5’ GTTCGAGCTCCGCAAGGCCTGGG 3’ |  |
| Plant 6 | 5’ GTTCGAGCTCCGCAA - GCCTGGG 3’ | biallelic |
|  | 5’ GTTCGAGCTCCGCAAAGTCCTGGG 3’ |  |
| *OsPIN5b*-site2 | 5’AACGTGTACGCCAGCGTTCTCGG 3’ |  |
| Plant 1 | 5’ (43-bp deletion)ACGGTGGGGCTGAAG 3’ | biallelic |
|  | 5’ (1-bp del)G(42-bp del)CGGTGGGGCTGAAG 3’ |  |
|  | 5’ CGGCGGCGGCGGCGTCCGGCTACCGTTCTGGGCGACGGCGAGGACGGTGG 3’ | Ref |
| Plant 13 | 5’ AAGGTGTACGCCAGCGTTCTCGG 3’ | heterozygous |
|  | 5’ AACGTGTACGCCAGCGTTCTCGG 3’ |  |
| Plant 6 | 5’ CCAGCGTTCTCGGCGTCGTGTGGGCGTGCATCGCG 3’ | heterozygous |
|  | 5’ CCAGCGTTCTCGGCGTCGTGGGGGCGTGCATCTCG 3’ |  |
|  | 5’ CCAGCGTTCTCGGCGTCGTGTGGGCGTGCATCGCGTACAGGTATATACGC  GTCAGCCATGCATTGCATGC 3’ | Ref |
| Plant 8 | 5’ GTGGGCGTGCATCGCGTACAAGTATATACGC 3’ | homozygous |
|  | 5’ GTGGGCGTGCATCGCGTACAGGTATATACGCGTCAG 3’ | Ref |
| Plant 24 | 5’ GTGGGCGTGCATCGCGTACACGTATATACGC 3’ | homozygous |
|  | 5’ GTGGGCGTGCATCGCGTACAGGTATATACGCGTCAG 3’ | Ref |
| *GS3*-site1 | 5’ GACGCGCTCCACCGCGAGATCGG 3’ |  |
| Plant 9 | 5’ (51-bp del) GTACAATCTATCTCTATC 3’ | homozygous |
|  | 5’ ACCGCCTCCAGCTCGCCGTCGACGCGCTCCACCGCGAGATCGG  ATTCCTCGAGGTACAATCTATC 3’ | Ref |
| Plant 21 | 5’ GACGCGCTCCACC - - GCGATCGG 3’ | homozygous |
| Plant 5 | 5’ GACGCGCTCCACC - - - - - ATCGG 3’ | homozygous |
| Plant 30 | 5’ GACGCGCTCCACC - - - - GATCGG 3’ | homozygous |
| Plant 6 | 5’ GACGCGCTCCAC - - - - AGATCGG 3’ | homozygous |
| *GS3*-site2 | 5’ CCTCCTCATGTGGGGCGGCGTTA 3’ |  |
| Plant 9 | 5’ CCTCCTACATGTGGGGCGGCGTTA 3’ | biallelic |
|  | 5’ CCTCCT - ATGTGGGGCGGCGTTA 3’ |  |
| Plant 21 | 5’ CCTCCTGCATGTGGGGCGGCGTTA 3’ | biallelic |
|  | 5’ CCTCCTTCATGTGGGGCGGCGTTA 3’ |  |
| Plant 27 | 5’ CCTCCTTCATGTGGGGCGGCGTTA 3’ | biallelic |
|  | 5’ CCTCCTACATGTGGGGCGGCGTTA 3’ |  |
| Plant 30 | 5’ CCTCCT - ATGTGGGGCGGCGTTA 3’ | biallelic |
|  | 5’ CCTCCTCCATGTGGGGCGGCGTTA 3’ |  |
| Plant 37 | 5’ CCTCCTACATGTGGGGCGGCGTTA 3’ | homozygous |
| *OsMYB30*-site1 | 5’ CCGGAGCAGTCGCTCTCGACCAC 3’ |  |
| Plant 7 | 5’ CCGGAGTCAGTCGCTCTCGACCAC 3’ | homozygous |
| Plant 11 | 5’ CCGGAG – AGTCGCTCTCGACCAC 3’ | biallelic |
|  | 5’ CCGGAGTCAGTCGCTCTCGACCAC 3’ |  |
| Plant 5 | 5’ CCGGAGGCAGTCGCTCTCGACCAC 3’ | biallelic |
|  | 5’ CCGGAGTCAGTCGCTCTCGACCAC 3’ |  |
| Plant 19 | 5’ (58-bp deletion)TTCCGACCACCGTGC 3’ | biallelic |
|  | 5’ CTCTCAACCA- - - - - - - - -CGACC 3’ |  |
|  | 5’ GCCGCGCCCAAGCGAAAGGCGACCAAGAAGGCTGCGGCGGTGGCGGTGGC 3’ | Ref |
| Plant 3 | 5’ (54-bp deletion)GACGTTCCGACCACC 3’ | biallelic |
|  | 5’ (1-bp del)C(53-bp del)ACGTTCCGACCACC 3’ |  |
|  | 5’ GCCGCGCCCAAGCGAAAGGCGACCAAGAAGGCTGCGGCGGTGGCGGTGGC 3’ | Ref |
| *OsMYB30*-site2 | 5’ CCGGTGGCATGCAGAATTTGCCC 3’ |  |
| Plant 7 | 5’ CCGGTGAGCATGCAGAATTTGCCC 3’ | biallelic |
|  | 5’ CCGGA – GCATGCAGAATTTGCCC 3’ |  |
| Plant 11 | 5’ AATTGGCCTCTTGGG (90-bp deletion) 3’ |  |
|  | 5’ (complicated variant) GGCTGGCCTCCTGCC 3’ |  |
|  | 5’ AATCTTTGGAGCTGTGTAAATCTTTACAAAGCGGAGAGATTGATGGGAAA 3’ | Ref |
| Plant 29 | 5’ TTGGCCTCTTGGGTC (88-bp deletion) 3’ |  |
|  | 5’ (complicated variant) CCGGCCCCTTGCGCG 3’ |  |
|  | 5’ AATCTTTGGAGCTGTGTAAATCTTTACAAAGCGGAGAGATTGATGGGAAA 3’ | Ref |
| Plant 27 | 5’ CCGGTG - - ATGCAGAATTTGCCC 3’ | biallelic |
|  | 5’ CCGGTGAGCATGCAGAATTTGCCC 3’ |  |
| Plant 6 | 5’ CCGGT - GCATGCAGAATTTGCCC 3’ | biallelic |
|  | 5’ CCGGTGAGCATGCAGAATTTGCCC 3’ |  |

Note: The PAM regions were highlighted in yellow and the edited bases were in red colour.

**Table S2 Primers used in this article**

| Primers | Primer sequence (5’-3’) |
| --- | --- |
| U6-OsMYB30-1F | GCCGGGCAAATTCTGCATGCCAC |
| U6-OsMYB30-1R | AAACGTGGCATGCAGAATTTGCC |
| U6-OsMYB30-2F | GCCGTGGTCGAGAGCGACTGCTC |
| U6-OsMYB30-2R | AAACGAGCAGTCGCTCTCGACCA |
| U6-OsPIN5b-1F | GCCGAACGTGTACGCCAGCGTTCT |
| U6-OsPIN5b-1R | AAACAGAACGCTGGCGTACACGTT |
| U6-OsPIN5b-2F | GCCGTTCGAGCTCCGCAAGGCCT |
| U6-OsPIN5b-2R | AAACAGGCCTTGCGGAGCTCGAA |
| U6-OsGS3-1F | GCCGTAACGCCGCCCCACATGAGG |
| U6-OsGS3-1R | AAACCCTCATGTGGGGCGGCGTTA |
| U6-OsGS3-2F | GCCGACGCGCTCCACCGCGAGAT |
| U6-OsGS3-2R | AAACATCTCGCGGTGGAGCGCGT |
| gRT#+u6-MYB30-1 | GGCAAATTCTGCATGCCACGTTTTAGAGCTAGAAAT |
| gRT#+u6-MYB30-2 | TGGTCGAGAGCGACTGCTCGTTTTAGAGCTAGAAAT |
| gRT#+u6- PIN5b-1 | AACGTGTACGCCAGCGTTCTGTTTTAGAGCTAGAAAT |
| gRT#+u6- PIN5b-2 | TTCGAGCTCCGCAAGGCCTGTTTTAGAGCTAGAAAT |
| gRT#+u6-GS3-1 | TAACGCCGCCCCACATGAGGGTTTTAGAGCTAGAAAT |
| gRT#+u6-GS3-2 | ACGCGCTCCACCGCGAGATGTTTTAGAGCTAGAAAT |
| OsU6aT# MYB30-1 | GTGGCATGCAGAATTTGCCGGCAGCCAAGCCAGCA |
| OsU6aT# MYB30-2 | GAGCAGTCGCTCTCGACCAGGCAGCCAAGCCAGCA |
| OsU6aT# PIN5b-1 | AGAACGCTGGCGTACACGTTGGCAGCCAAGCCAGCA |
| OsU6aT# PIN5b-2 | AGGCCTTGCGGAGCTCGAAGGCAGCCAAGCCAGCA |
| OsU6aT# GS3-1 | CCTCATGTGGGGCGGCGTTAGGCAGCCAAGCCAGCA |
| OsU6aT# GS3-2 | ATCTCGCGGTGGAGCGCGTGGCAGCCAAGCCAGCA |
| UF | CTCCGTTTTACCTGTGGAATCG |
| GR-R | CGGAGGAAAATTCCATCCAC |
| Pps-R | TTCAGA*GGTCTC*TACCGACTAGTATGGAATCGGCAGCAAAGG |
| Pgs-2 | AGCGTG*GGTCTC*GTCAGGGTCCATCCACTCCAAGCTC |
| Pps-2 | TTCAGA*GGTCTC*TCTGACACTGGAATCGGCAGCAAAGG |
| Pgs-3 | AGCGTG*GGTCTC*GTCTTCACTCCATCCACTCCAAGCTC |
| Pps-3 | TTCAGA*GGTCTC*TAAGACTTTGGAATCGGCAGCAAAGG |
| Pgs-4 | AGCGTG*GGTCTC*GAGTCCTTTCCATCCACTCCAAGCTC |
| Pps-4 | TTCAGA*GGTCTC*TGACTACATGGAATCGGCAGCAAAGG |
| Pgs-5 | AGCGTG*GGTCTC*GGTCCACATCCATCCACTCCAAGCTC |
| Pps-5 | TTCAGA*GGTCTC*TGGACTTGTGGAATCGGCAGCAAAGG |
| Pgs-6 | AGCGTG*GGTCTC*GCAGATAGTCCATCCACTCCAAGCTC |
| Pps-6 | TTCAGA*GGTCTC*TTCTGCAATGGAATCGGCAGCAAAGG |
| Pgs-L | AGCGTG*GGTCTC*GCTCGACGCGTATCCATCCACTCCAAGCTC |
| myb30-f | CGCCGTGCTGCGAGAAGA |
| myb30-r | TTAGACTAAAGTGTTATGACTTTAAAACCACC |
| PIN5b-f | ATGGCGCCGCTGTACTTCG |
| PIN5b-r | ACTTTTCGAACTGGGTCGAAAACAC |
| GS3-1f | CTTGACTACTCGTTGGAAGTGTGCG |
| GS3-1r | ACGGGCCCAGAGGAAGAAG |
| GS3-2f | GCCAAATTGTGTTAAATTTCAAATAATTT |
| GS3-2r | GTAAAATAAATCAGCAATCACGTACTCA |
| Off site 1f | AAGTGGAACTGAGGGCCTTC |
| Off site 1r | GCTTAATCATGTTGTTGAACAACG |
| Off site 2f | ATGATTGACCTTTTGACTTTCAGTC |
| Off site 2r | GGCGTGCAAGGAGCGCA |
| Off site 3f | AAGGGATGAGGGATGTGAAATAGAC |
| Off site 3r | GAAGGTAAGGTAAAGAGATAAGGTGTGGG |
| Off site 4f | GCGAGCTGCTCCGCATTG |
| Off site 4r | CCTCCCCTCCAGGTGCG |
| Off site 5f | GGCATGACCTCCCCTCTTTTTTG |
| Off site 5r | CGTGAAAATGATGTGGATGGTGATGA |
| Off site 6f | TGCGCAACTCCGTGATCG |
| Off site 6r | CGTAGTGCTCGACCCCCG |
| Off site 7f | CAAAAGAGGCATTCGGTTTTG |
| Off site 7r | CTCGTCGTCCTCCACCTCC |
| Off site 8f | CCCTCGACACCCGACTCCCTCCAT |
| Off site 8r | CGTGGTTGTTGTGTGCTTTTGTGGTG |
| Off site 9f | ATGATCTCACGGTGGTTGACGGT |
| Off site 9r | CATGACCCGATCGTCGCCCC |
| Off site 10f | CCGGCGGGAGAAGGAATG |
| Off site 10r | GGATGGATGGATGGACGG |
| Off site 11f | TGGAGAAAGAGAGAGAGAGAGAGAGAGAGAGAGAG |
| Off site 11r | CCATAGCAACAAAGCTCAGGCCCT |
| Off site 12f | CAAACTTTACAAACGCGTATACAAAC |
| Off site 12r | AATTGAGGGACCCAAAGTGAAC |
| YUC1-RTf | AGGTGTTGGTCGTGGGATGCG |
| YUC1-RTr | GCGATGCCGAACGTGGATAGA |
| BMY10-RTf | CTTCCGCGCCTCGTCG |
| BMY10-RTr | AATGGTGTCCCGATGGCG |
| CYCT1-RTf | GCATTTGTTGCAGCTCAAG |
| CYCT1-RTr | TCACCACTTCGCTGACTTATTG |
| H1-RTf | GCAAGGCACCTGCAGCTT |
| H1-RTr | AGGCAGCCTTTGTACAGATCCT |
| SP-l | GCGGTGTCATCTATGTTACTAG |
| SP-r | TGCAATAACTTCGTATAGGCT |
| Cas9-f | CTGACGCTAACCTCGACAAG |
| Cas9-r | CCGATCTAGTAACATAGATGACACC |

Note: The italic sequence represents *Bsa* I enzyme restriction site.
